# Resource implications of preparing individual participant data from a clinical trial to share with external researchers

**DOI:** 10.1186/s13063-017-2067-4

**Published:** 2017-07-17

**Authors:** Catrin Tudur Smith, Sarah Nevitt, Duncan Appelbe, Richard Appleton, Pete Dixon, Janet Harrison, Anthony Marson, Paula Williamson, Elizabeth Tremain

**Affiliations:** 1 Department of Biostatistics, University of Liverpool, Block F, Waterhouse Building, 1-5 Brownlow Street, Liverpool, L69 3GL UK; 2 Alder Hey Children’s NHS Foundation Trust, Liverpool, UK; 3 Department of Molecular and Clinical Pharmacology, University of Liverpool, Liverpool, UK; 4 National Institute for Health Research Evaluation, Trials and Studies Coordinating Centre, University of Southampton, Southampton, UK

**Keywords:** Individual participant data, IPD, Data sharing, Clinical trial, Anonymisation, Transparency, Cost

## Abstract

**Background:**

Demands are increasingly being made for clinical trialists to actively share individual participant data (IPD) collected from clinical trials using responsible methods that protect the confidentiality and privacy of clinical trial participants. Clinical trialists, particularly those receiving public funding, are often concerned about the additional time and money that data-sharing activities will require, but few published empirical data are available to help inform these decisions. We sought to evaluate the activity and resources required to prepare anonymised IPD from a clinical trial in anticipation of a future data-sharing request.

**Methods:**

Data from two UK publicly funded clinical trials were used for this exercise: 2437 participants with epilepsy recruited from 90 hospital outpatient clinics in the SANAD trial and 146 children with neuro-developmental problems recruited from 18 hospitals in the MENDS trial. We calculated the time and resources required to prepare each anonymised dataset and assemble a data pack ready for sharing.

**Results:**

The older SANAD trial (published 2007) required 50 hours of staff time with a total estimated associated cost of £3185 whilst the more recently completed MENDS trial (published 2012) required 39.5 hours of staff time with total estimated associated cost of £2540.

**Conclusions:**

Clinical trial researchers, funders and sponsors should consider appropriate resourcing and allow reasonable time for preparing IPD ready for subsequent sharing. This process would be most efficient if prospectively built into the standard operational design and conduct of a clinical trial. Further empirical examples exploring the resource requirements in other settings is recommended.

**Trial registration:**

SANAD: International Standard Randomised Controlled Trials Registry: ISRCTN38354748. Registered on 25 April 2003. MENDS: EU Clinical Trials Register Eudract 2006-004025-28. Registered on 16 May 2007. International Standard Randomised Controlled Trials Registry: ISRCTN05534585/MREC 07/MRE08/43. Registered on 26 January 2007.

## What’s new

- Clinical trial data sharing is beneficial and commonly encouraged.
- Preparing appropriately anonymised datasets, associated documentation and relevant information requires planning and resources.
- In this exercise the data pack preparation process has been outlined and the time and cost of preparing data from two publicly funded clinical trials has been estimated.
- Clinical trial researchers, funders and sponsors can use these empirical estimates to guide future discussions and planning, and determine the appropriate allocation of resources.

## Background

The need to increase transparency and efficiency in medical research has led to trial funders, medical journals, regulators, pharmaceutical companies and academic researchers devoting substantial attention to the topic of sharing individual participant data (IPD) from clinical trials. Numerous data-sharing platforms or portals such as “Clinical Study Data Request” [1], “YODA” [2], “BioLINCC” [3] and “Project Data Sphere” [4] have been established in an attempt to improve access to IPD from clinical trials across a wide range of trial designs and disease areas. Many journals, including NIHR Journals Library, Annals of Internal Medicine, BMJ, and BMC Medicine, encourage data sharing and require a formal statement describing the conditions under which raw data are accessible [5]. A recent proposal by the International Committee for Medical Journal Editors, ongoing work by the European Medicines Agency (EMA), and the introduction of a new item on data sharing within trial registry information by ClinicalTrials.gov are set to further change the culture, expectations and attitudes towards sharing IPD. Although data sharing is not a new concept in this field, the procedures and practicalities of implementation are being re-examined and responsibilities rearticulated as part of the drive for transparency.

Guidance has been developed [6–8] to raise awareness, emphasise good practice and to encourage the development of transparent processes to facilitate responsible sharing of IPD from clinical trials. To date, there has been a general preference towards sharing clinical trial IPD within controlled access systems [9, 10], which offer a mechanism to safeguard the integrity and scientific validity of subsequent data use whilst maximising the protection of trial participants’ privacy. Before sharing the data, a series of steps would need to be followed, which may include identifying relevant datasets and documentation, verifying any data-sharing restrictions from the wording of participant consent forms, anonymising participant-level data, preparing trial documentation and a data dictionary and preparing data-use agreements.

Concern about the additional resources required is a commonly cited barrier to sharing IPD from clinical trials [11–13], particularly for publicly funded clinical trials [9]. Experience from industry-sponsored clinical trials suggests that an average of 7 days would be required to redact documentation, prepare anonymised data, co-ordinate data access proposals and load datasets to a sharing platform (Robert Frost, personal communication). However, little is known about the resource implications for sharing IPD from publicly funded trials. In this paper we summarise the steps involved and evaluate the resources required for preparing anonymised IPD and associated documentation ready for sharing, from two different publicly funded clinical trials.

## Methods

Two publicly funded clinical trials coordinated by the University of Liverpool were identified to be used in this exercise. 

The SANAD (Standard And  New Antiepileptic Drugs; a randomised controlled trial examining the longer-term outcomes of standard versus newer anti-epileptic drugs) trial​ [14–16], recruited and followed patients between 1999 and 2006 and was initiated prior to Medicines for Human Use (Clinical Trials) Regulations 2004 [17]. SANAD was funded by a £1.35 million grant from the National Institute for Health Research (NIHR) Health Technology Assessment (HTA) Programme, with an additional 20% of resource contributed from pharmaceutical companies with products assessed in the trial. However, the funding sources had no role in study design, data collection or analysis and interpretation of data. SANAD was a relatively large trial including 90 centres, 5 drugs and 2437 participants. The methods of data collection and storage were robust, but information systems were quite different to the current systems used by the Clinical Trials Research Centre (CTRC) at the University of Liverpool. Data were collected on paper case report forms (CRFs) and entered onto a central database in Microsoft Access.

The MENDS (The use of MElatonin in children with Neuro-developmental Disorders and impaired Sleep; a randomised, double-blind, placebo-controlled, parallel study) trial [18, 19], also funded by the NIHR HTA programme with a £1 m grant, is a randomised double-masked placebo-controlled trial that randomised 146 children with neuro-developmental problems from across 18 centres to two intervention groups, between 2007 and 2010. At each clinic and home visit the research practitioner entered IPD directly onto a laptop with contents securely synchronised with a central InferMed MACRO (version 3) database, including a full audit trail of data changes.

In this exercise, a statistician took the lead in locating and preparing the datasets and documentation for each trial and it was assumed that IPD from a trial would be shared using a controlled access system with a suitable approval process and data-use agreement in place to help safeguard trial participants’ confidentiality. The participant consent form used in each trial was examined to identify any specific data-sharing restrictions. Datasets were anonymised by creating a summary of variables within each dataset (if not already available), identifying and removing operational variables such as those related to data queries or validations, identifying direct and indirect patient identifiers and applying anonymisation rules based on recent work undertaken by the PHUSE group [20]. Briefly, the process comprises recoding unique identifying numbers (such as subject ID), offsetting dates or converting dates to relative study day and removing or recoding personally identifiable information, potentially sensitive information and consideration of the risk of re-identification from extreme values, rare characteristics and from verbatim free text. A description of the anonymisation method used for each variable was created.

**Fig. 1:**
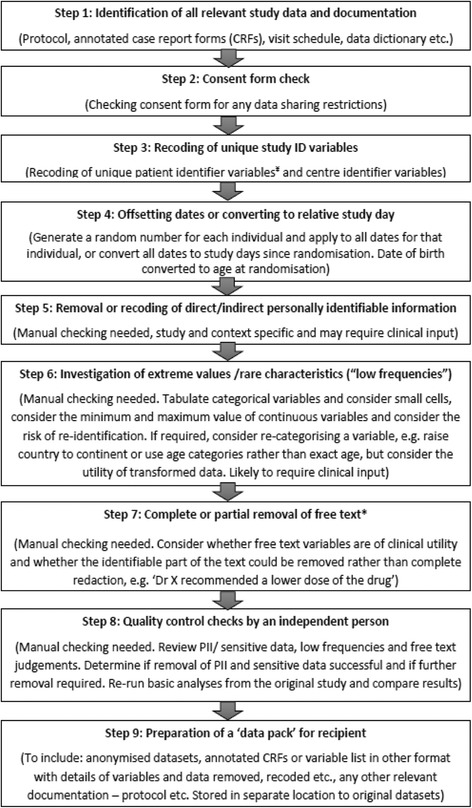
Steps involved in the anonymisation process. ^¥^The unique patient identifier code and date of randomisation can provide valuable information about the sequence and pattern of randomisation. Recoded data should be supplemented by complete flow of trial participants, highlighting any randomisation errors. *Steps *4* to *7* could be performed in any order. *PII* personally identifiable information, *CRF* case report form

Variable recoding, removal and redaction were undertaken by developing code within an SAS statistical analysis programme. Following completion of the anonymisation process, an independent statistician checked the datasets to verify that all relevant direct and indirect participant identifiers had been anonymised appropriately, as would be required prior to releasing the final anonymised data to external researchers. The contents of a final “data pack”, including relevant documentation and anonymised datasets, were transferred to a new secure folder, separated from all original trial-related information, where access could be securely provided to outside researchers. A summary of the steps involved is provided in Fig. 1 and the previously recommended contents of a data pack have been summarised in Table 1 (see reference [8] for further details).

**Table 1:** Suggested content of the data pack ready for sharing

Suggested content of data pack	Description
Anonymised data	Electronic data collected for each patient in the trial in a format that can be recognised by a wide range of statistical software (e.g. SAS, Stata, R). The use of “StatTransfer” or other similar product may be useful for this purpose
Blank CRF	Blank CRFs with descriptions of the data collected. These could be annotated to provide a map of the data variables within the dataset, or provided as blank CRFs along with dataset specifications
Dataset specifications	Meta-data describing the datasets e.g. data-freeze date, variable labels, variable descriptions, formats, anonymisation method applied to each variable and summary of amendments made during the trial e.g. changing data definitions, adding/removing variables
Protocol	Trial protocol, including all amendments
Statistical analysis plan	Methods of analysis and procedures for data handling used in the final statistical analysis (this is useful if researchers want to replicate published analyses to facilitate their understanding of the dataset)
Analysis programs	Programmes used for generating and analysing data used in the final analysis report (this is useful if researchers want to replicate published analyses to facilitate their understanding of the dataset)
Clinical study report (CSR) (or equivalent) if applicable	Report of efficacy and safety data from the trial that forms the basis of submissions to regulatory authorities e.g. EMA

*CRF* case report form, *EMA* European Medicines Agency

The number of hours and trial team members involved with executing each step of the process was recorded. The cost associated with preparing each data pack was estimated by assuming an average salary for each trial team member and applying this to the relevant number of hours required to perform each step. Archiving costs were also estimated and included in these calculations. Final project costs were estimated based on a “full economic cost” model that would be relevant if data packs were being prepared by clinical trial units supported by higher education establishments in the UK.

## Results

### SANAD

There was no formal summary of variables (no annotated CRF, dataset specification or data dictionary) available to accompany the trial dataset, which included a total of 387 unique variables of which 98 were coded as text variables. Several variables were identified as direct and indirect patient identifiers, some of which would not commonly be collected in current trials e.g. patient names, address and General Practitioner (GP) contact details. The data on quality of life had been collected and managed by a separate research group and the dataset was stored in a separate location to the clinical dataset.

As the SANAD trial had been completed more than 10 years ago, the trial team had been disbanded and obtaining access to all datasets and all relevant documentation took 10.5 hours in total. Data had been entered onto a database using a version of Microsoft Access that was now obsolete, and additional time was required to access and export the data to a suitable format to allow anonymisation to be undertaken. A blank copy of the lengthy quality-of-life patient questionnaires had only been stored in hard copy paper format, and additional time was required to transfer these to an electronic format ready for sharing.

It took a total of 32 hours for the statistician to generate anonymised datasets and create a data dictionary to accompany the datasets to explain variable names and formats and to summarise whether individual variables had been recoded, removed or redacted. Identifying variables and applying the relevant anonymisation rule was a very manual process, complicated by the fact that the format of some variables had been incorrectly defined within the Access database, e.g. date variables incorrectly defined as character variables. Judgements had to be made as to whether particular text variables contained information that could potentially be used to re-identify patients, which again was a lengthy and manual process.

The statistician then took 1.5 hours to prepare the data pack containing the relevant documentation and anonymised datasets, which were transferred to a secure folder. The data pack was accessed by an independent statistician who checked that the datasets could be understood, had been appropriately anonymised and did not contain data that could be used to re-identify trial participants. This final quality control and validation step took 6 hours.

In total, it took 50 hours and involved a statistician, a member of the information systems (IS) team and a trial manager to prepare the data pack for the SANAD trial (Table 2).

**Table 2:** Time required to prepare the data pack for the SANAD trial

Step of process	Role	Tasks	Time (hours)
Getting access to the data and documentation	Statistician	• Requesting access and liaising with TM and IS • Accessing secure folder to open datasets (some problems encountered when trying to access shared folder) • Establishing relevant data tables e.g. table summarising payments made to GPs would not be relevant • Setting up secure shared folder	2.5
	Information systems	• Working out how to open Access database (old version) • Exporting tables from Access database to SAS	3
	Trial manager	• Locating data files and documentation • Transferring to secure shared drive • Scanning blank CRFs and paper copy of the blank quality-of-life patient questionnaire	5
De-identification	Statistician	• First stage of de-identification (IDs, personally identifiable information and dates), preparation of variable list and identification of free-text variables • Second stage of de-identification: redaction of free text, generation of aggregated tables and preparation of variable list. Judgements made regarding what could be redacted and variables kept, redacted and tabulated or redacted completely	32
Final data pack	Statistician	• Pull all relevant files and documentation together and transfer to separate secure folder	1.5
Quality control check	Statistician	• Independent statistician to understand the datasets and check through to ensure that relevant data have been de-identified and check any remaining text variables are suitably redacted to protect patient privacy	6
Total	Statistician		42
	Information systems		3
	Trial manager		5
	Overall		50

*TM* trial manager, IS information systems, *GP* General Practitioner, *CRF* case report form

### MENDS

A full data dictionary was available listing the definition and description of each data variable and the collection of personally identifiable information had been restricted. The MENDS dataset included 650 unique variables, of which 150 were coded as text variables. Data entered were synchronised with a central InferMed MACRO (version 3) database; although MACRO databases are still used within the CTRC (currently version 4), version 3 requires different processes and systems to those currently used to extract trial data and documentation from the MACRO database. Also, the statistician performing anonymisation was not involved in the original trial and was not familiar with MACRO databases, so obtaining access to all datasets and all relevant documentation took 7.5 hours of statistician and IS time in total.

It took a total of 26 hours for the statistician to generate anonymised datasets and create a data dictionary to accompany the datasets to explain variable names and formats and to summarise whether individual variables had been recoded, removed or redacted. The majority of the 150 text variables were free text, mainly describing reported reasons why patients had not taken medication or had not completed sleep diaries. In addition to personally identifiable information such as patient or treating clinician names or initials, such free text often made references to times of year (e.g. forgot to take medication as away on summer holidays; disturbed sleep as child was excited for Christmas). Due to the potential for inconsistency between offset dates and dates described in free text, and the risk of re-identification, the decision was taken to completely redact all free-text variables. It may be possible to provide aggregated information from these free-text variables (e.g. a table of reasons for missed doses of medication) to researchers on request if required.

The statistician then took 1 hour to prepare the data pack containing the relevant documentation and anonymised datasets, which were transferred to a secure folder. Final quality control and validation was carried out as described previously; this step took 5 hours. 

In total it took 39.5 hours and involved a statistician and member of IS staff to prepare the data pack for the MENDS trial. A breakdown of the steps involved is shown in Table 3.

**Table 3:** Time required to prepare the data pack for the MENDS trial

Step of process	Role	Tasks	Time (hours)
Getting access to the data and documentation	Statistician	• Requesting access • Understanding layout of datasets and format • Understanding how to extract into SAS (statistician had not used these systems before)	1.5
	Information systems	• Setting up access to SQL server tables • Setting up access to MACRO data • Providing documentation about the tables, questions groups and visits • Providing documentation of the visit schedule • Providing a list of variables in MACRO • Providing details of logins and website access MACRO	6
De-identification	Statistician	• First stage of anonymisation (IDs, PII and dates), preparation of variable list and identification of free-text variables • Second stage of anonymisation: redaction of free text, generation of aggregated tables and preparation of variable list. • Judgements had to be made regarding what could be redacted and variables kept, redacted and tabulated or redacted completely. Further redaction may be needed where times of year are referred to in free text (e.g. Christmas, Easter).	26
Final data pack	Statistician	• Pull all relevant files and documentation together and transfer to separate secure folder	1
Quality control check	Statistician	• Independent statistician to check the datasets to ensure that relevant data have been anonymised and check any remaining text variables are suitably redacted to protect patient privacy	5
Total	Statistician		33.5
	Information systems		6
	Overall		39.5

*PII* personally identifiable information

### Cost of data pack preparation

The cost of data pack preparation was estimated as £3185 for SANAD and £2540 for MENDS assuming full economic costs. A full breakdown is shown in Table 4.

**Table 4:** Estimated cost of data pack preparation

Staff role^a^	Approximate salary (£)	SANAD cost (£)	MENDS cost (£)
Senior statistician	56,482	911	717
Junior statistician	31,342	506	398
Senior IS staff	44,620	51	103
Junior IS staff	37,394	43	86
Senior trial coordinator	44,620	86	0
Junior trial coordinator	31,342	60	0
Archiving	34,233	93	93
Total directly incurred staff (£)		1750	1397
Estimate of full economic cost		1435	1143
Total project cost		3185	2540

*IS* information systems. ^a^Assumed a 50:50 split in contribution between senior and junior staff where applicable

## Discussion

We assumed that data were being shared with other trusted researchers and that this was being done within the confines of a controlled access system whereby a research proposal had already been reviewed and approved and a data use agreement had been signed by relevant parties. These measures provide additional assurance that the risk of re-identification of patients has been minimised and that the data are being shared in a responsible way. However, administering requests and processing data-use agreements will require additional resources that have not been considered during this exercise. Increased efficiency is likely to be gained if data requests, approval and access are coordinated through a centralised multi-sponsor system such as clinicalstudydatarequest.com [1] and YODA [2].

We have assumed that the data being shared are clean and ready to analyse. However, it is possible that queries may arise about the data following data release and some additional resource may be necessary to address those. Neither of the trials in this exercise had used data standards and this is fairly typical of other publicly funded trials co-ordinated by similar trials units in the UK [9]. However, we recognise that the pharmaceutical industry does use standards such as CDISC, and it is likely that more publicly funded trials will adopt such approaches in the future. We believe that this will only help to improve the efficiency of data preparation and subsequent future analyses that combine IPD from multiple studies. We also did not explore the resources that may be required to undertake additional motivated intruder tests, which may be necessary if dealing with particularly sensitive information [21].

The resource requirements are likely to be greater for older legacy trials as the documentation and datasets may take longer to locate, and the process of navigating the datasets to enable anonymisation may be more challenging for individuals that were not involved in the trial originally. Wherever possible, data pack preparation should therefore be undertaken at the end of the trial by individuals with knowledge of the trial datasets [6, 8] and this process should be incorporated as a standard step during the conduct of a trial. This is more efficient and will also ensure that any potential issues or concerns about the data are appropriately recorded to facilitate greater understanding. Future data sharing should also be considered during the design of a trial with the collection of unnecessary data, or potentially identifying text variables, avoided wherever possible if appropriate, and plans for data sharing included within the protocol and data management plan [6].

Whilst we have completed this exercise with a statistician taking the lead in data preparation activities, we recognise that in practice statistical expertise may be scarce and could be better directed towards working on the development of new trial projects with the responsibility for data preparation shifted towards the data management and information systems teams if capacity allows. There may be further efficiency gains from automating some of the process. In addition, as trialists and clinical trial units become more experienced at preparing data and the research community share experiences to help improve the efficiency of data sharing, the resources required as outlined in this exercise may well decrease in the future.

Data sharing should be prospectively incorporated into the operational plan, design and conduct of a clinical trial. Organisations that fund and sponsor clinical trials have significant leverage to set standards and to encourage data sharing for the trials they support [7]. However, there is a need for funders and sponsors to acknowledge that data sharing activities will require additional resources. The exercise presented in this manuscript provides reasonable initial estimates to guide decisions. However, a major limitation is that estimates are based only on experience from two clinical trials conducted in similar settings. This is unlikely to be representative of all trials and the resources required for data preparation may differ in other settings necessitating the need for further empirical evidence to inform future decisions. Furthermore, it is important to recognise that different clinical trials units adopt different approaches to project management and some variability in costings is inevitable. Nevertheless, it is comforting to see that the time and cost of data preparation will generally be small compared to the overall cost of a trial, such that costs, if pre-specified adequately, should not constitute a major obstacle to data sharing in the future. Similarly, organisations that provide infrastructure for coordinating clinical trials should acknowledge that resources are required to process and administer requests for data, and further empirical research is required to investigate the cost of this aspect of data sharing.

## Conclusions

During this exercise we have estimated the resource requirements for preparing anonymised IPD, from two publicly funded clinical trials, ready for sharing. The process of preparing a dataset and documentation ready for sharing took approximately 50 hours, at a total cost of £3185 for the SANAD trial, and approximately 39.5 hours, at a total cost of £2540 for the MENDS trial. The following recommendations are made:

1. Funding for preparing data for sharing should be incorporated into grant applications for publicly funded clinical trials.
2. Preparing data packs will likely require input from statistics and information systems staff. It may also be necessary to involve clinical, trial management and data management input. One individual should be given the role of leading the data pack preparation and liaising with other relevant individuals.
3. Involving information systems staff to develop automated systems to redact patient identifying information, and de-identify data during the data extract, could be more efficient than statisticians developing programmes to do this.
4. Clinical trial database developers should consider the possibility of including automated anonymisation tools in future versions.
5. The data pack should be prepared at the end of the trial by individuals with knowledge of the trial datasets. This is more efficient and will also ensure that any potential issues or concerns about the data are appropriately recorded for any future data users.

## Abbreviations

CRF: Case report form
CTRC: Clinical Trials Research Centre
EMA: European Medicines Agency
GP: General Practitioner
HTA: Health Technology Assessment
IPD: Individual participant data
IS: Information systems
MENDS: The use of melatonin in children with neuro-developmental disorders and impaired sleep; a randomised double-blind, placebo-controlled, parallel study
NIHR: National Institute for Health Research
PHUSE: Pharmaceutical Users Software Exchange
PII: Personally identifiable information
SANAD: Standard and new antiepileptic drugs; a randomised controlled trial examining the longer-term outcomes of standard versus newer anti-epileptic drugs
SAS: Statistical Analysis System
YODA: Yale University open data access project
